# Population genetic structure of European wildcats inhabiting the area between the Dinaric Alps and the Scardo-Pindic mountains

**DOI:** 10.1038/s41598-021-97401-5

**Published:** 2021-09-09

**Authors:** Felicita Urzi, Nikica Šprem, Hubert Potočnik, Magda Sindičić, Dean Konjević, Duško Ćirović, Andrea Rezić, Luka Duniš, Dime Melovski, Elena Buzan

**Affiliations:** 1 Faculty of Mathematics, Natural Sciences and Information Technologies, University of Primorska, 6000 Koper, Slovenia; 2 Faculty of Agriculture, Department of Fisheries, Apiculture, Wildlife Management and Special Zoology, University of Zagreb, 10000 Zagreb, Croatia; 3 Biotechnical Faculty, University of Ljubljana, 1000 Ljubljana, Slovenia; 4 Faculty of Veterinary Medicine, University of Zagreb, 10000 Zagreb, Croatia; 5 Faculty of Biology, University of Belgrade, 11000 Belgrade, Serbia; 6 Georg-August University Goettingen, Wildlife Sciences, 37073 Göttingen, Germany; 7 Macedonian Ecological Society, Skopje, 1000 Republic of Macedonia; 8 Environmental Protection College, 3320 Velenje, Slovenia

**Keywords:** Genetic hybridization, Population genetics

## Abstract

Habitat fragmentation and loss have contributed significantly to the demographic decline of European wildcat populations and hybridization with domestic cats poses a threat to the loss of genetic purity of the species. In this study we used microsatellite markers to analyse genetic variation and structure of the wildcat populations from the area between the Dinaric Alps and the Scardo-Pindic mountains in Slovenia, Croatia, Serbia and North Macedonia. We also investigated hybridisation between populations of wildcats and domestic cats in the area. One hundred and thirteen samples from free-leaving European wildcats and thirty-two samples from domestic cats were analysed. Allelic richness across populations ranged from 3.61 to 3.98. The observed Ho values ranged between 0.57 and 0.71. The global F_ST_ value for the four populations was 0.080 (95% CI 0.056–0.109) and differed significantly from zero (*P* < 0.001). The highest F_ST_ value was observed between the populations North Macedonia and Slovenia and the lowest between Slovenia and Croatia. We also found a signal for the existence of isolation by distance between populations. Our results showed that wildcats are divided in two genetic clusters largely consistent with a geographic division into a genetically diverse northern group (Slovenia, Croatia) and genetically eroded south-eastern group (Serbia, N. Macedonia). Hybridisation rate between wildcats and domestic cats varied between 13% and 52% across the regions.

## Introduction

According to a revised taxonomy the European wildcat is classified into two subspecies *Felis silvestris silvestris Schreber*, 1777 and *Felis silvestris caucasica Satunin*, 1905, distributed in European forest habitats including islands of Britain, Sicily and Crete^1^. The species is legally protected by both Bern Convention^2^ and European Union’s Habitats Directive, which consider it "strictly protected"^3^. International Union for Conservation of Nature (IUCN), categorized wildcat as “Least Concern”, since it has been evaluated together with *Felis lybica* species distributed over vast regions of Asia and Africa, and without considering the demographic decline and fragmentation of European wildcat populations^4^.

Hundreds of years of combined negative factors, including habitat loss, have resulted in the extinction of the European wildcat from most of its former range in many parts of Europe^5^. In addition, transport networks, urban areas as well as agricultural landscapes divide natural habitats into small isolated patches and create barriers that restrict gene flow and ultimately leads to a hidden genetic structure within the European wildcat populations^4,6–8^. Many recent studies^9–12^ showed that wildcat populations are geographically structured and conservation strategies should improve gene flow by restoring ecological corridors within biogeographical units^13,14^. Human-induced mortality and disease transmission^7,15–17^ are also important threats to wildcats in Europe^5,18^, but the loss of genetic purity due to hybridisation with domestic cats (*Felis catus*) (i.e. the introgression of some alleles present in domestic cats into the genotype of the wildcats)^4,19–21^ is a threat that has attracted the most attention from the scientific community and the public.

Hybridisation between wildcats and domestic cats can lead to (i) disruption of local genetic adaptations, (ii) loss of genetic integrity of the European wildcats and even extinction of the subspecies^22^. Introgression of artificially selected traits of domestic cats into species of conservation concern may affects their fitness and leads to outbreeding depression in wild populations^23^. The extent of gene flow from domestic cats to wildcats varies in intensity across Europe and may exhibit significant local differences, most likely based on historical or ecological traits^24–27^. For example, a high level of hybridisation has been observed in Scottish^24^, and Hungarian^26^ wildcats, while a low hybridisation rate was found in sampled wildcats from Italy, Bulgaria, Portugal, and Germany^9,28,29^. Identifying areas with different levels of the domestic cat gene introgression in European wildcat populations could help recognizing factors that have facilitated introgression rates in the past and/or that currently hinder or accelerate hybridisation. Since the level of hybridisation appears to be low in some regions and high in others, it is likely that other factors, such as differences in habitat structure and behaviour, have played a role in reducing hybridisation^9^.

According to the genetic analysis, the European wildcats are subdivided into five main phylogeographic clusters, each corresponding to five biogeographic groups, distributed in the Iberian Peninsula, Italian Peninsula and the region of Sicily, Central Europe, Central Germany and Northern Balkans (Dinaric Alps)^11,28^. These geographically distinct groups represent the living remains of the Pleistocene refugial population^30^. More detailed analyses within each of the phylogeographic clusters could clarify the current patterns of structuring within population, since a possible influence of the "refugia within the refugia" existed throughout the Pleistocene^31^. On the other hand, recent habitat loss and fragmentation have led to population bottlenecks and a reduction in genetic diversity^26^. But despite the awareness of the importance of gaining more insight into the underlying patterns of genetic variability and genetic integrity of local populations, data on population structure are lacking in several European regions. The work of Mattucci et al.^11^, which included 39 samples from the area of Slovenian Dinaric Alps, but did not include any samples of wildcat populations of Balkan Peninsula, underlines the importance of clarifying evolutionary history of the wildcats in this area.

In this study we used microsatellite markers to analyse genetic variation and structure of the wildcat populations from north-western Dinaric Alps to the Scardo-Pindic mountain system. Regardless they probably originate from the same Pleistocene refugium, we investigated whether geographical isolation is reflected in the genetic structure of wildcat populations. Finally, we investigated hybridisation between populations of wildcats and domestic cats.

## Results

### Genetic variation between wildcat populations

A total of 20 loci (18 used for analysis) on 145 individuals (113 wildcats and 32 domestic cats) from four countries were examined (treated as "populations", Table 1). Two loci (FCA090 and FCA094) were hard to read and were therefore excluded from all analyses. All 18 microsatellites were polymorphic, showing from seven (FCA058, FCA149) to 18 (FCA096) alleles per locus. The independent replication of 10% of the samples provided no evidence for false alleles. The allele sizes differed in the expected multiples of the microsatellite repeats. Eight out of 72 comparisons of loci by sample location deviated significantly from the expectations of Hardy–Weinberg (*P* < 0.05). In the 18 microsatellite loci (with less than 5% of null alleles in all populations) the average null alleles frequencies per locus ranged from zero (FCA096) to 0.091 (FCA088) with an average of 0.033. No identical genotypes were observed, the low values for probability of identity (PID) suggest that individuals in the study were not highly related: PID = 7.8 × 10^–19^, PIDsibs = 1.2 ×  × 10^–7^ in wildcats; PID = 3.7 × 10^–21^, PIDsibs = 3.2 × 10^–8^ in domestic cats.

**Table 1:** Genetic diversity among European wildcat populations in the area between the Dinaric Alps and the Scardo-Pindic Mts. based on 18 microsatellite loci.

Country	ab	N	Hybrids	He ± SD	Ho ± SD	F_IS_	HWE	A ± SD	AR ± SD	PID	PIDsibs
Slovenia	SI	22	3	0.685 ± 0.199	0.697 ± 0.237	− 0.019	0.664	5.39 ± 1.76	3.61 ± 0.91	3.2 × 10^–16^	5.4 × 10^–07^
Croatia	HR	55	9	0.724 ± 0.196	0.715 ± 0.203	0.013	**0.044**	7.55 ± 2.20	3.98 ± 0.91	7.8 × 10^–19^	1.2 × 10^–07^
Serbia	SR	29	15	0.694 ± 0.221	0.658 ± 0.229	0.054	0.181	5.78 ± 2.01	3.84 ± 1.12	6.7 × 10^–17^	4.6 × 10^–07^
N. Macedonia	MK	7	1	0.692 ± 0.203	0.570 ± 0.265	**0.191**	**0.004**	4.28 ± 1.32	3.74 ± 1.05	6.3 × 10^–15^	1.4 × 10^–06^
Croatia (domestic)	DcHR	32	–	0.783 ± 0.207	0.697 ± 0.273	**0.110**	** < 0.001**	8.66 ± 1.88	4.43 ± 0.69	3.7 × 10 ^-21^	3.2 × 10^–08^

Standard deviations are for average values; P < 0.05 for HWE and F_IS_ (before Bonferroni correction) is indicated in bold He expected heterozygosity, Ho observed heterozygosity, F_IS_ inbreeding coefficient, HWE Hardy–Weinberg equilibrium, A number of alleles, AR allelic richness (calculated by the rarefaction method for the lowest sample size n = 10), PID: probability-of-identity.

The populations of MK and HR showed significant deviations from HWE based on exact tests in GENEPOP (*P* < 0.05), additionally the population from MK showed deviation also based on F_IS_ (significantly positive values). It can be expected that the deviation from HWE in MK population is a consequence of the small sample size included in the analysis. The HR population showed no significant deviation from HWE after the Bonferroni correction (Table 1). The number of alleles per locus in wildcats ranged from 2 to 18 with a mean of 6.77. Allelic richness across populations ranged from 3.61 to 3.98, with the highest values in HR and SR populations. A similar pattern was observed for Ho with values between 0.57 and 0.71 and He with values between 0.68 and 0.72, with MK population showing the lowest Ho.

The global F_ST_ value for the four populations was 0.080 (95% CI 0.056–0.109) and differed significantly from zero (*P* < 0.001). The pairwise F_ST_ values between populations ranged from 0.004 to 0.148 with the mean of the pairwise F_ST_ = 0.070 ± 0.060 (± *SD*) and also differed significantly from zero (*P* < 0.05) (Table 2). The highest F_ST_ value was observed between the populations MK and SI and the lowest between SI and HR.

**Table 2:** Pairwise values of F_ST_ among European wildcat populations and domestic cats.

Population	Slovenia	Croatia	Serbia	North Macedonia
Croatia	0.004			
Serbia	0.025	0.019		
North Macedonia	0.050	0.028	0.018	
Domestic	0.147	0.127	0.148	0.136

All F_ST_ values are significant.

### Exclusion of hybrids individuals

#### Detection of simulated hybrids with NewHybrids

All controls were correctly identified by the NEWHYBRIDS software, with posterior probabilities of 0.99 for wildcats and 0.95 for domestic cats. The results of the identification of genotypes of the simulated hybrids are presented in the Supplementary Fig. 1, panel a1, b1, c1.

#### Detection and exclusion of hybrids

STRUCTURE and NEWHYBRIDS analysis concordantly identified two samples with genotypes of domestic cats and 25% (*n* = 28) of hybrids across all wildcat samples using the exclusion criterion given by a z value of less than 0.85 (NEWHYBRIDS) and a q value of less than 0.80 (STRUCTURE). Numbers of hybrids varied across countries: SI 13% (*n* = 3), HR 16% (*n* = 9), SR 52% (*n* = 15), MK 14% (*n* = 1). Nineteen of the 28 hybrids were classified as F2 hybrid (SI, *n* = 3; HR, *n* = 4; SR, *n* = 12), four were classified as back-crosses of F1 to wildcat (HR, *n* = 2; SR, *n* = 2), four as back-crosses of F1 to domestic cat (HR, *n* = 3; SR, *n* = 1) and two as domestic cats P2 population (SI, *n* = 1; SR, *n* = 1) (see Supplementary Fig. 1, panel a2, b2, c2).

### Genetic and spatial clustering

**Figure 1:**
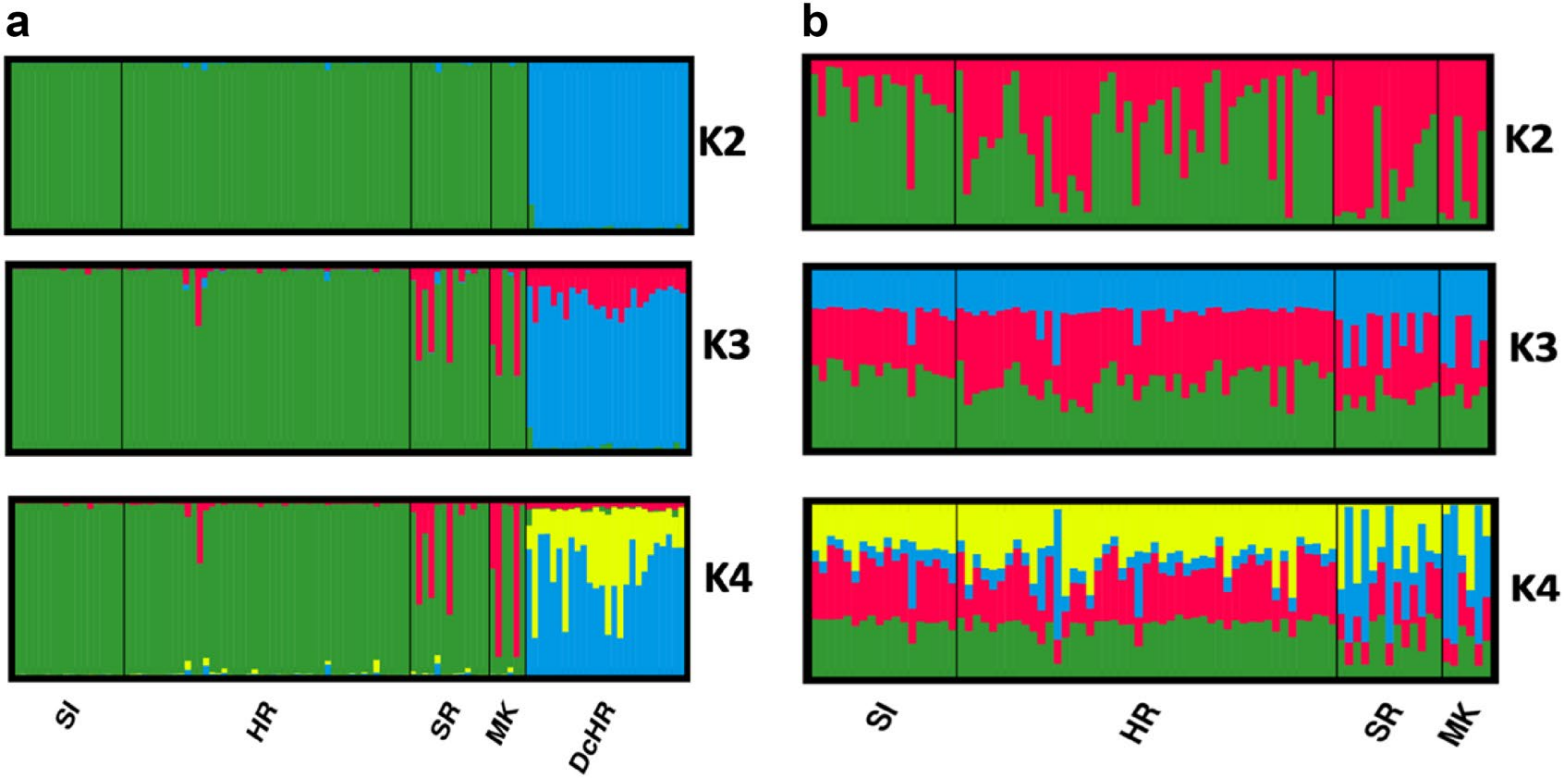
Genetic structure of European wildcat populations from Slovenia (SI), Croatia (HR), Serbia (SR), North Macedonia (MK) and domestic cats (DcHR)) (**a**) and only European wildcat populations (**b**) revealed by STRUCTURE) (see Table 4.) Each individual is represented by a line proportionally partitioned into colour segments corresponding to its membership in particular clusters. K is the number of clusters. Black lines separate the individuals from different populations (according to Table 4).

The STRUCTURE analysis clearly separated the European wildcat samples from domestic cats (K = 2; Fig. 1a). The estimated probability value for each K indicate the smallest value at K = 3 (see Supplementary Fig. 2). Increasing K to 3 split the European wildcats into two subclusters, separating populations SI and HR from populations SR and MK; no additional structuring was found at K = 4.

**Figure 2:**
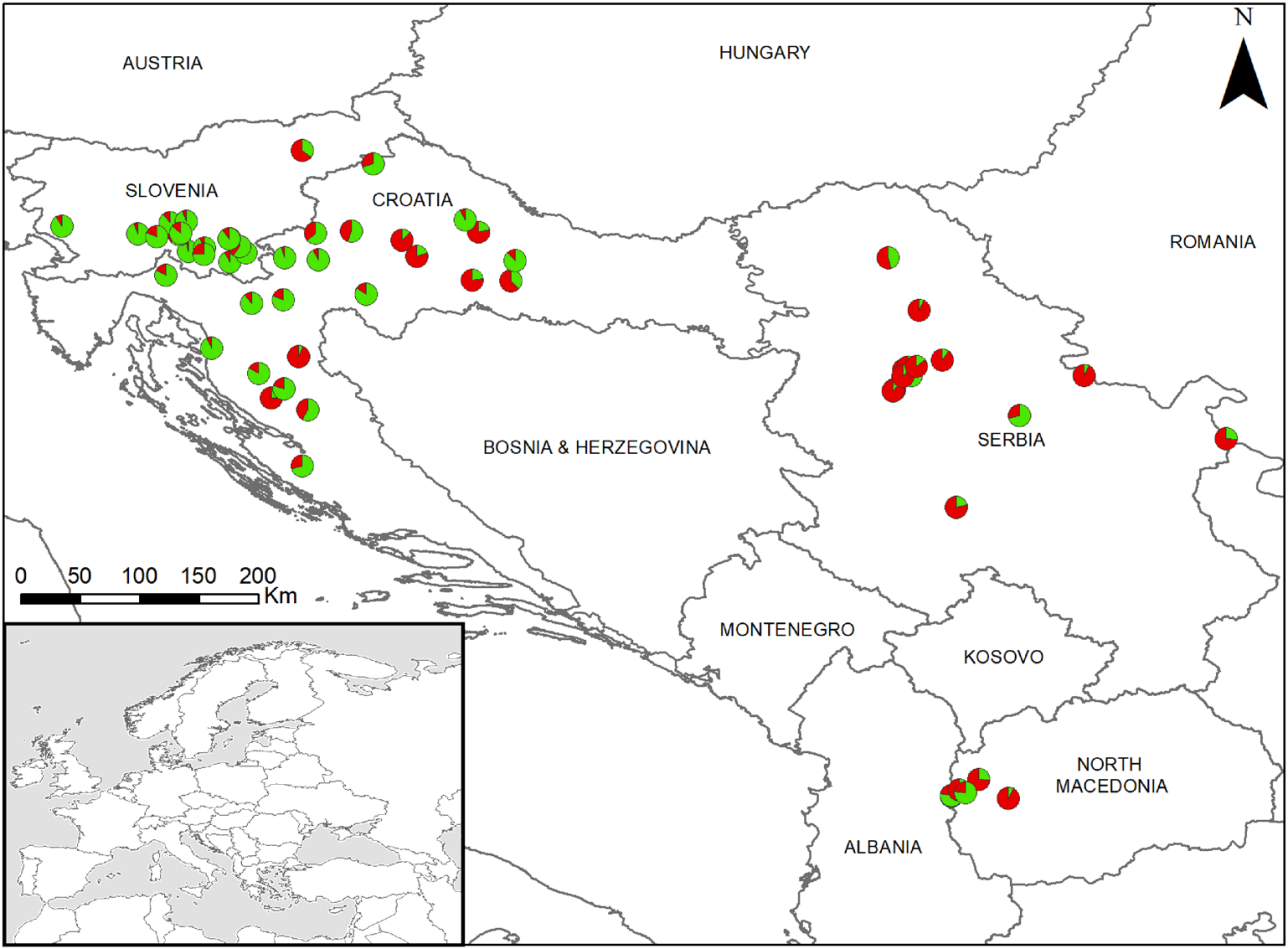
Genetic structure of European wildcats inhabiting the area between the Dinaric Alps and the Scardo-Pindic mountains based on spatial clustering of individuals according to the best model, dividing four populations into two clusters (K = 2; see also Fig. 1b).

In the separate analysis performed only with the wildcat samples, the highest ΔK values were obtained with K = 2 (Fig. 1b), suggesting a division between western and south-eastern populations (Fig. 2). For the logarithm probability of K it was possible to observe the lowest value of K = 2 that captures the maximum degree of structure detected in the data (see Supplementary Fig. 3). K = 3 and K = 4 showed no further difference in geographic structuring, suggesting that the two wildcat clusters are largely consistent with a geographic division into a northern group (SI, HR) and a south-eastern group (SR, MK) (Fig. 1b).

**Figure 3:**
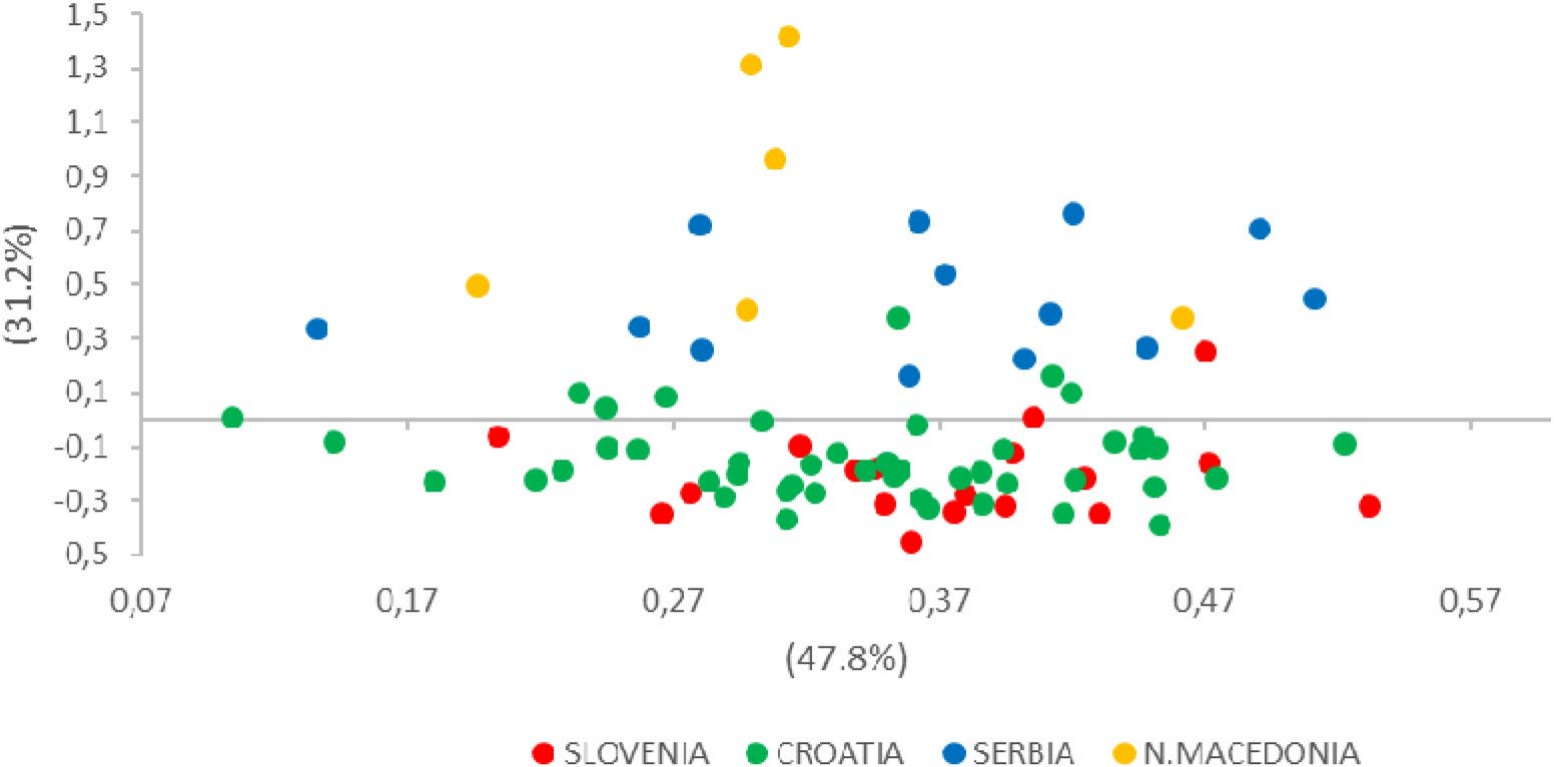
A two-dimensional plot of the FCA performed using GENETIX. European wildcats from different populations are indicated by different colours. The first axis explained 47.8% (*P* = 0.010), and the second explained 31.2% (*P* = 0.072) of the variance.

The FCA plot, which was based on individual genotypes, clearly separated individuals along the second axis and two main groups were identified according to their rough geographical origin. The first factorial axis explained 47.8% of the variance within populations. Along the second axis, SI and HR populations were separated from SR and MK populations (Fig. 3).

DAPC according to the Bayesian Information Criterion, which also includes domestic cat genotypes, indicated that there are two genetic clusters of wildcats that distinguish individuals according to their geographical origin (in north–south gradient). Domestic cats belong to an independent cluster. The first principal component distinguished between domestic cats and wildcat clusters, and the second principal component showed a distinction between two wildcat clusters (see Supplementary Fig. 4). The ellipses, which describe the spatial extension of the clusters, did not overlap, which indicates a strong genetic structuring.

The AMOVA result highly supported group structuring revealed by STRUCTURE, DAPC and FCA, the variance was 1.47 and significant (*P* < 0.001) (Table 3).

**Table 3:** Hierarchical analysis of molecular variance (AMOVA) based on microsatellite data for European wildcat populations.

Source of variation	Variance
Among populations	0.69 (0.076)
Within population	2.01 (0.112)
Among group	**1.47** (< 0.001)

Values in bold are significant (P < 0.001). The populations correspond to four populations defined by country (according to Table 4), groups correspond to the two clusters according to the result of FCA and STRUCTURE analysis (K = 2). P values are given in parenthesis.

#### Isolation by distance

**Figure 4:**
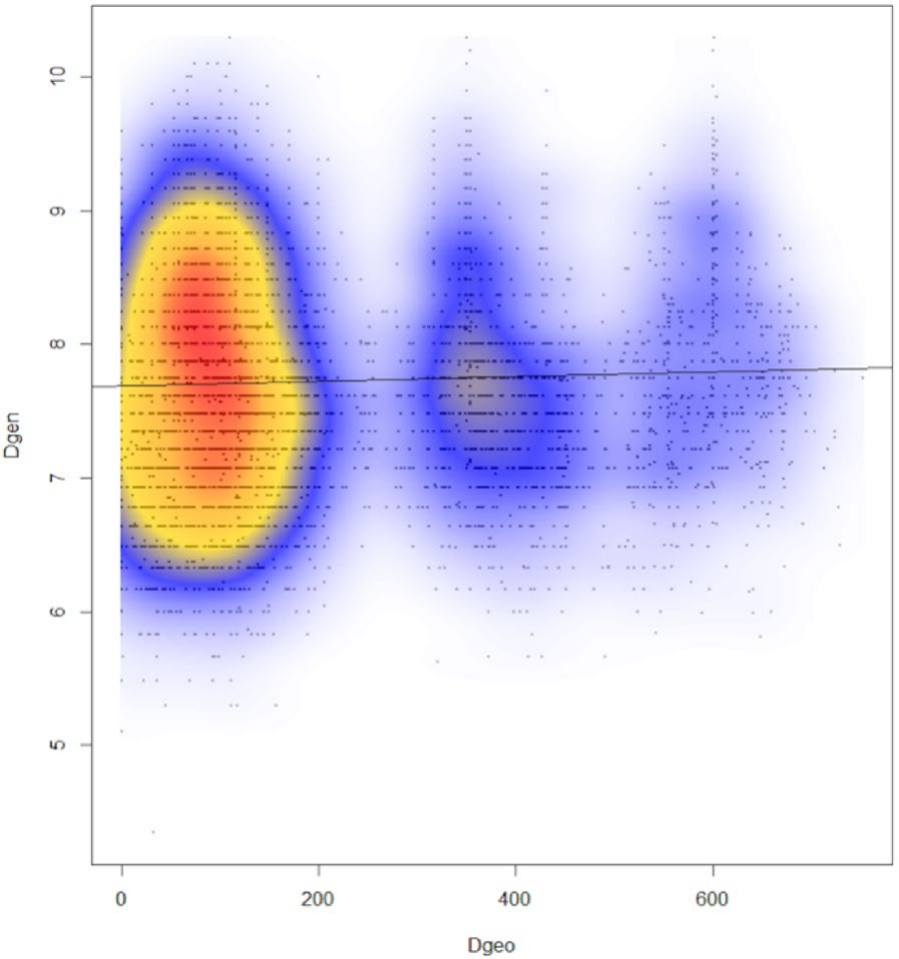
Isolation by distance. Pairwise Edwards genetic distances between individuals (Dgen), plotted against the Euclidean geographical distances (Dgeo; km) for the same individuals. Local density of points plotted using a two-dimensional Kernel density.

Microsatellite based genetic distances were correlated with geographical distances among populations (*t-value* = 3.012, *P* = 0.003, *R*^2^ = 0.0013), supporting the hypothesis of isolation by distance (Fig. 4).

## Discussion

By utilizing microsatellites, we have determined the genetic variation and population structure in the European wildcats inhabiting area between the Dinaric Alps, the Pannonian Plain and the Scardo–Pindic mountains. In this way, we contribute to the knowledge of the wildcat populations’ genetics in SE Europe, where data on the genetic outlook of this endangered felid are completely lacking. But genetic data are not the only one lacking. Both historical and recent data on wildcat distribution, abundance, mortality and other ecological factors that might affect genetic structure are missing for most countries included in our study. Mortality data is available only for Slovenia, for the 1950–1990 period, when annual culling varied between 70 and 493 individuals with an average of 193 wildcats culled per year^32^. In 1970s, based on hunters’ observations, Slovenian Hunting Association estimated the population size on up to 1000 individuals, mostly distributed in the sub-Mediterranean and Dinaric karst with occasional occurrence in the northern areas of Slovenia^32,33^. According to the Croatian Hunting Association, the estimated population size of the wildcat in Croatia is less than 2000 animals. Other demographic data is not available for any of the countries, so our results really do provide one of the rare insights into ecology of the species in this area.

Across our study area we found a slightly higher observed heterozygosity (Ho = 0.57–0.71) than reported in the study by Matucci et al.^11^, where the Ho across Europe ranged between 0.58–0.63. However, this value varies greatly among countries; e.g. central Germany 0.50–0.79^34^, France 0.39–1.00^35^, Portugal 0.42–0.82^29^, Hungary 0.42–0.87^26^. By implementing appropriate conservation measures with avoidance of increased introgression at the national level, we expect that genetic diversity in SI and HR populations could be sufficient to maintain adequate variation for adaptive evolution, especially given the observed gene flow between countries. High diversity was also confirmed with mitochondrial DNA control region of wildcats from Croatia^36^. On the contrary signs of genetic erosion were observed in both SR and MK populations. In Serbia we found relatively high diversity, but greater F_IS_ value compared to the SI and CR populations and very high level of introgression of domestic alleles. SR wildcat population is fragmented and occupies patches of suitable habitats along wooded river banks in the northern part and forest habitat patches in the central and southern parts of the country which may present barriers to gene flow and consequently affect genetic integrity. Thus, additional studies are needed to reveal fine-scale genetic structure in this area. Lower genetic diversity that we found among wildcats sampled in MK should be considered with caution due to the low sample size but could be an indication of recent genetic bottlenecks or geographic isolation due to various human impacts^5,37–39^.

The highest F_ST_ values in our study were observed between the northern SI population and the southern MK population, and lowest between the closest SI and HR population, which is in congruent with geographical distances. We found a signal for the existence of isolation by distance (Fig. 4) between populations, while AMOVA data indicate the existence of two genetic groups in which the SI and HR populations overlap. The existing admixture among them is reflected by a weak pattern of isolation by distance and congruent results for the population divisions obtained by FCA, DAPC and STRUCTURE.

Although a high dispersal potential of the wildcat has been demonstrated^34,40^, a small-scale genetic structure within the regions could be a consequence of the limited use of wildlife corridors due to natural landscape barriers in the study area^41^. It is also possible that habitat fragmentation in a more urbanized part of the Dinaric region contributed to the lower connectivity^42^ and subsequent F_ST_ values found within this area. It has also been shown in central Germany that anthropogenic and natural landscape barriers can limit the wildcat's dispersal potential and the consequences are reflected in the genome^34^. Our findings indicate that habitat between SI and HR is continuous and barely limits gene flow for the wildcats, but it is difficult to draw conclusion considering SR and MK population. All populations were not evenly sampled, we did not analyse samples from eastern part of Croatia, so we have a sampling gap between Croatia and Serbia along the Pannonian Basin. Also, due to ad-hoc sampling we have quite low coverage in Serbia and very scarce in North Macedonia, which might affect values of genetic differentiation^43^.

Furthermore, our northern and south-eastern populations might display the recolonisation from different refugia. A model of late Pleistocene isolation and genetic diversification of European wildcat populations into three main Mediterranean glacial refuges in the southern Iberian, Italian, and Balkan peninsulas was proposed by Hewitt^44^ and Mattucci et al.^11^. Authors also state that this genetic divergence cannot be explained by recent fragmentation, but only by prolonged periods of isolation with historically no or limited gene flow^10,11^. Mattucci et al.^10^ suggest a scenario of ancient population isolation in Alpine (Italy, Slovenia) and Mediterranean (Istrian peninsula; south west of Slovenia and Croatia) refuges which increase genetic divergence between European wildcats in the Eastern Alps and Apennines. They also showed that the European wildcat populations are subdivided into at least five main biogeographic groups with divergence times from the Late Pleistocene, but sampling in Dinaric Alps, Pannonian Plain and Scardo—Pindic mountains was scarce (only samples from Slovenia and Bosnia and Herzegovina were included in the study)^11^. So Mattucci et al.^11^ already noted that STRUCTURE results suggest that local populations could be genetically subdivided at smaller geographical scale but additional genetic markers analysis is needed to point us into this direction. Studies on Eurasian lynx, the only other wild Felidae present in this area, showed that the mitochondrial lineage restricted to the Balkan lynx population was the first one to diverge from the other Eurasian lynx haplogroups. Balkan lynx haplogroup was the most divergent and its split was dated around 96.6 kya^45^, while the three Mediterranean wildcat lineages probably originated 21,000–125,000 years ago^11^. Past population isolation in Balkan refuges with genetically diverse groups of populations, leading to a more recent ‘refugia within refugia’ concept as also seen in other species (e.g. wild boar)^46^. Lack of information on the demographic history of the researched populations and gaps in our sampling prevent us from drawing firm conclusions, so additional studies based on fine-grained grid sampling are needed to reveal fine-scale genetic structure in this area.

Our analyses are in line with previous studies showing that the genetic integrity of the European wildcat is not compromised in some regions of Europe^11,27^, however in some areas introgression can be substantial^21,26^ and could be caused by environmental circumstances that enable long-lasting hybridisation^24,26,47^. In our study none of the hybrids found were classified as hybrids of the first generation which shows low level of recent hybridisation in all studied populations. In SI and SR mainly second-generation hybrids were found, while in HR a half of hybrids were classified as back-crosses and a half as second-generation hybrids. Overall, 25% of the sampled wildcats were admixed, which is comparable to the observation in a wildcat population in the Swiss Jura (21%)^21^. In the case of SR, due to high hybridisation rate, this result could reflect a long-lasting hybridisation that persists in the area and might be the consequence of high density of stray domestic cats, especially on the periphery of remote village areas. By using genomic approaches Mattucci et al.^28^ found that some of the hybrids with possible F2 origins actually showed admixture traces dating back from 9 to 11 generations in the past. Since F2 hybrids are expected to occur in rare cases, a high proportion of F2 hybrids in the SI and SR populations could be the result of method bias and brings misassignments of individuals that can represent the product of repeated crosses among F1 or F2 individuals, rather than true second-generation hybrids. We used STRUCTURE and NEWHYBRIDS for classifying the hybrids. But most likely, the highly polymorphic, non-diagnostic genetic markers we have used are not accurate enough to classify the hybrids with repeated crossbreeding between different hybrid generations. Results of Q scores generated by STRUCTURE allow for a relatively simplistic tracking of estimated proportion of ancestry, so that backcrosses cannot be distinguished from more complex hybrids. Even if NEWHYBRIDS classifies the hybrids into generational categories, some misassignments of individuals could occur due to strict hybrid categories used in this approach.

In the area from the Dinaric Alps to the Scardo-Pindic Mts. wildcats are divided in two genetic clusters largely consistent with a geographic division into a genetically diverse northern group (SI, HR) and genetically eroded south-eastern group (SR, MK). But wider sampling, especially in MK and neighbouring countries, like Bosnia and Herzegovina, would help to clarify the evolutionary history in this part of Europe. The apparent loss of genetic integrity due to hybridisation with domestic cats found in Serbia urges specific conservation measures to maintain evolutionary potential of this species. Hybridization with domestic cats is considered the greatest threat to wildcats in several countries^4,24,47^, and the need for practical measures that could be relevant to conservation has been highlighted^48^. The simulation project in the Swiss Jura shows that reducing the number of domestic cats and hybrids and improving the quality and quantity of habitats by supporting the highest possible wildcat densities are priorities for wildcat conservation^48^.

## Methods

### Study area

The study area, across four countries (Slovenia, Croatia, Serbia and North Macedonia) is located at the intersection of three major European geographical units, namely Dinaric Alps, Pannonian Basin, and the Scardo-Pindic Mts. system, which is a continuation of Dinarides in the southern part of Balkan Peninsula.

The two-mountain systems, Dinaric Alps (extending from Slovenia to Albania) and Scardo-Pindic Mts. (from Kosovo to Greece) are characterized by small plateaus and meadows at high altitude up to 2,764 m a.s.l. The forest cover consists mainly of beech (*Fagus sylvatica*), fir (*Abies alba*) and spruce (*Picea abies*) associations^49^. The climate varies, but roughly with a continental climate in the north-east Pannonian region, a severe alpine climate in the mountain regions and a sub-Mediterranean climate in the coastal region along the Adriatic Sea^50^. Topographically, the area is highly heterogeneous, interrupted by ditches, bays and rocks developed on limestone and dolomite rocks. Besides wildcat, several other carnivorous species are present in the region; brown bear (*Ursus arctos*)*,* grey wolf (*Canis lupus*), Eurasian lynx (*Lynx lynx*), golden jackal (*Canis aureus*), red fox (*Vulpes vulpes*), and several species of mustelids (*Mustelidae*). Unlike the mountainous regions, there are no large carnivorous species in the Pannonian region, only mesocarnivores, but there are areas with increasing populations of the golden jackal.

Management of wildcats is somewhat different in each country and is governed by national conservation policy (see Table 4).

**Table 4:** Location, sample size, number of detected hybrids and brief history of European wildcat populations inhabiting the area between the Dinaric Alps and the Scardo-Pindic Mts.

Country	ab	N	Hybrids	Historical Management	National status (census)	Brief description of populations
Slovenia	SI	22	3	Hunted until the 1992. There is no management plan and no coordinated monitoring	Protected (1000)	Large population occupying optimal habitats in Dinaric Mts. Size reduced in the 1970, recovered afterwards. Distributed in Alps, Dinaric Mts. and small Pannonian areas in the northeast of the country
Croatia	HR	55	9	Hunted until 2013. Before 2013 hunting was only allowed in areas north of Sava river. There is no management plan and no coordinated monitoring	Protected (< 2000)	Lack of data regarding the history of population. Distributed in all suitable habitats from Dinaric Mts. to Pannonian region
Serbia	SR	29	5	Still game species. In the province Vojvodina hunted until the 90 s. There is no management plan and no national monitoring	Protected in north province Vojvodina (no data available for population size)	In the north of Vojvodina province, the distribution is associated with wooded river banks (rivers Danube, Tisa, Begej and Tamiš). In the southern area of Vojvodina (the entire Srem region and southeast of the Banat) and south of rivers Sava and Danube wildcat occurs in forest habitats
North Macedonia	MK	7	1	Hunted until 2009.There is no management plan and no coordinated monitoring	Protected from 2009 (no data available for population size)	No available data

### Sampling

A total of 113 samples from free-leaving putative European wildcats were collected over a period from 2012 to 2020 in Slovenia (SI), Croatia (HR), Serbia (SR) and North Macedonia (MK) (see Supplementary information Fig. 5) from dead (legal hunting, natural mortality and vehicle collisions) or from live-trapped individuals for telemetry studies (Table 4). They were morphologically identified as wildcats by collectors. Blood samples from domestic cats were taken in Croatia (32 samples in total) from animals admitted for treatment at University Hospital of Faculty of Veterinary Medicine, University of Zagreb. All samples were stored at − 80 °C, tissue samples in 95% ethanol, whole blood samples in sodium citrate vacutainer. Based on the country of origin, the wildcat individuals were grouped into four groups (for the purposes of this paper further considered as "populations").

### DNA extraction and microsatellite genotyping

Genomic DNA was extracted using the peqGOLD Blood & Tissue DNA Mini Kit (VWR International Ltd., Leuven, Belgium) according to manufacturer’s instructions. The concentration and purity of the extracted DNA in the final elution volume was measured with Qubit® dsDNA BR Assay Kit (Invitrogen) on 3.0 Qubit Fluorometer (Life Technologies). Nineteen autosomal dinucleotide and one tetranucleotide (FCA 441) microsatellites (Supplementary Table S1), originally identified in domestic cats^51^ and screened in studies in wildcats and domestic cats^10,11,52^, were amplified in six PCR multiplex reactions with ready-to-use KAPA2G Fast Multiplex Mix (Kapa Biosystems). According to the manufacturer's instructions we used 2 µL template DNA and 0.3 mM final concentration for each primer used in the set. The amplification was performed under the following conditions: initial PCR activation for 3 min at 95 °C, followed by 35 cycles of denaturation for 15 s at 95 °C, annealing for 30 s at 58 °C, extension for 30 s at 72 °C and final extension for 10 min at 72 °C. The fragment analysis was performed on a SeqStudio sequencer (Thermo Fisher Scientific) using the GeneScan LIZ500 (−250) standard (Applied Biosystems). The results were validated with the software GENEMAPPER v.5.0 (Applied Biosystems). Negative controls were included in all extraction and PCR steps. About 10% of the randomly selected samples were replicated independently to check for false alleles.

### Analyses of genetic variation

Using the FREENA program^53^, we estimated the proportion of the null allele (NA) at each locus in each population with respect to the fact that the presence of null alleles can cause a significant heterozygote deficit and deviation from Hardy–Weinberg equilibrium (HWE). We used the software GENEPOP 4.2^54^ to test for deviations from HWE. The exact test to assess the heterozygosity deficiency was performed for each population (country). The baseline significance level was set at 0.05 and a Bonferroni procedure was applied in multiple comparisons to compensate for the risk of a bloated type 1 deficiency.

The mean number of alleles (A), observed (H_O_) and expected (H_E_) heterozygosity^55^, and inbreeding coefficients (F_IS_) were calculated for each population with GENETIX 4.05.2^56^ separately for the domestic, and wildcat populations. The probability of identity and sibling-identity were calculated with the Excel macro GenAlEx v6.5^57^. Allelic richness for each population (AR) was estimated following a rarefaction method in the program FSTAT 2.9.4^58^. The genetic differentiation between wildcat populations and between domestic cats and wildcats was estimated using pairwise F_ST_ in GENEPOP 4.2 according to Weir & Cockerham^59^, and significant differences from zero F_ST_ estimates were tested with 1,000 permutations. All subsequent analyses were performed after excluding individuals with admixed genotypes (see paragraph “Control population for hybrid simulation”).

### Exclusion of hybrids individuals

#### Determination of control population for hybrid simulation

Based on the initial STRUCTURE analysis, there was a statistically supported split between wildcat and domestic cat clusters (Fig. 1a). Ten individuals from each of our four population (SI, HR, SR, MK) and ten individuals from HR domestic cat population was selected for the control population used in the simulation of hybridisation (to obtain clear hybrid genotypes). The controls for pure parental wildcats (P1) and domestic cat (P2) were determined above (*z* designation) by NEWHYBRIDS v1.1^60^. These *z* values were used to determine the ten animals from each population that were the “most pure”, with the *z* value above 0.99. These individuals were used as control animals for each population in subsequent analyses.

#### Simulated hybrids

One hundred simulated genotypes (for each population independently) were generated from the control populations P1 and P2 in HYBRIDLAB v1.0^61^ for: parental wildcats (P1) and domestic cat populations (P2), F1, F2, back-crosses of F1 to wildcat (P1Bx) and domestic cat (P2Bx) and a second back-cross of P1Bx to wildcat controls (P1Bx2) and P2Bx to domestic cat controls (P2Bx2).

The simulated genotypes were analysed using NEWHYBRIDS. A burn- in period of 5 000 was followed by 10 000 sweeps based on the graphical version of NEWHYBRIDS (see Supplementary Fig. 1, panel a1, b1, c1). Ten replicates using Jeffrey’s priors were tested and summarized using CLUMPAK. These simulated data were also analysed using STRUCTURE with K values of one to four with 100 000 burn-in and a data collection of 100 000 chains. The “Admixture Model” was applied. This protocol was replicated 10 times per each K-value. STRUCTURE HARVESTER was used to evaluate which K-value was most likely. The results of the replicated runs were combined with the Greedy algorithm of CLUMPP and the summary outputs were graphically displayed using RStudio and R version 3.6.2^62^.

#### Detection of hybrids

All 113 complete genotypes of putative wildcats were analysed with NEWHYBRIDS. A burn-in period of 5 000 was followed by 10 000 sweeps. Ten replicates using Jeffrey’s priors were tested. CLUMPAK was used to summarise the ten replicates for each prior. Individuals with a *z* designation value lower than 0.85 for either P1 or P2 were classified as a hybrid. Hybrids were further assigned to F1, F2, P1Bx or P2Bx, based on admixture analyses of observed and simulated cat data sets.

In addition, we used program STRUCTURE (as described by Mattucci et al.^10^) to compare the results of hybrids identification by NEWHYBRIDS; the admixed genotypes were identified at a threshold qi < 0.80. All individuals with assigned admixed genotype using NEWHYBRIDS and STRUCTURE methods were removed from the data sets.

#### Genetic and spatial clustering after hybrids exclusion

Population genetic clusters were revealed using STRUCTURE 2.3.4^63^ on two datasets: (i) wildcat populations and domestic cats, and (ii) wildcat populations only. In STRUCTURE, ten independent runs were performed for each K-value in the range of one to ten using a model assuming admixture with correlated allele frequencies. Each run included a burn-in period of 100,000 replications followed by 100,000 Markov Chain Monte Carlo (MCMC) iterations. The results of the replicated runs for each K value from two to ten were combined using STRUCTURE HARVESTER v 0.6.94^64^ and the optimal K value was selected using the ΔK method developed by Evanno et al.,^65^. The results of the replicated runs for the optimal K value were combined using the Greedy algorithm of CLUMPP 1.1.1^66^ and the summary results were plotted with DISTRUCT 1.1^67^.

The genetic relationships between all genotyped individuals were represented by Factorial Correspondence Analysis (FCA) using GENETIX. The distribution of the individuals in a 2D space was compared by eye with the geographical location of the localities. We also investigated the genetic structure with the R-package, adegenet 2.0.0^68^, using RStudio^69^ and R version 3.6.2^62^. We used the Discriminant Analysis of Principal Components (DAPC)^70^, multivariate method in this package to identify the most likely number of clusters (K).

The analysis of molecular variance (AMOVA)^71^ was performed in ARLEQUIN 3.5.^72^ to test the genetic differences between individuals and populations and between the optimal number of clusters identified by STRUCTURE (K = 2). The statistical significance of the variance components was investigated using 999 permutations in the R-package ade4 v1.7–13. ^73^. Finally, we tested isolation by distance (IBD) patterns within all populations using the Mantel function in the R-package adegenet.

### Ethical statements

Samples of European wildcats were collected from dead (legal hunting, natural mortality and vehicle collisions) or from live-trapped individuals for telemetry studies.

All methods were carried out in accordance with the Ethical and Welfare Standards presented in the (Official Gazette of the Republic of Croatia 102/2017), Regulation on the Protection of Animals Used for Scientific Purposes (Official Gazette of the Republic of Croatia 55/13), with the approval of the Bioethical Committee for the Protection and Welfare of Animals of the University of Zagreb Faculty of Agriculture (UR.BR. 251-71-29-02/19-21-2).

The study is reported in accordance with ARRIVE guidelines^74^.

## Supplementary Information

Supplementary Information.

## Data Availability

The data that support the findings of this study are available from the corresponding author upon reasonable request.
