# Supplementary material for: Population genetic structure of European wildcats inhabiting the area between the Dinaric Alps and the Scardo-Pindic mountains
Source: Sci Rep. 2021 Sep 9;11:17984. doi: 10.1038/s41598-021-97401-5 (PMC8429547; doi:10.1038/s41598-021-97401-5)
Supplement: Supplementary file 1 — Supplementary Information. [file 41598_2021_97401_MOESM1_ESM.docx]

**Supplementary Table**

**Table S1.** Description of 20 autosomal microsatellite loci used for genotyping of Felis silvestris samples

| **Multiplex** | **Locus_ID** | **Primer** |  | **Label** | **Allele size** |
| --- | --- | --- | --- | --- | --- |
| M1 | FCA058 | FCA058-F | CATCCCTGACTAGCCTGAGC | NED | 118-234 |
|  |  | FCA058-R | GTGAAGAAAGCTGGTGTGCA |  |  |
|  | FCA077 | FCA077-F | GGCACCTATAACTACCAGTGTGA | VIC | 132-162 |
|  |  | FCA077-R | ATCTCTGGGGAAATAAATTTTGG |  |  |
|  | FCA088 | FCA088-F | AGGAAAATGAAGTCAAGAAAATGG | FAM | 99-125 |
|  |  | FCA088-R | TTTTTCTTTTTCCCGTAATACACA |  |  |
| M2 | FCA008 | FCA008-F | ACTGTAAATTTCTGAGCTGGCC | VIC | 144-160 |
|  |  | FCA008-R | TGACAGACTGTTCTGGGTATGG |  |  |
|  | FCA149 | FCA149-F | CCTATCAAAGTTCTCACCAAATCA | PET | 116-134 |
|  |  | FCA149-R | GTCTCACCATGTGTGGGATG |  |  |
|  | FCA026 | FCA026-F | GGAGCCCTTAGAGTCATGCA | NED | 126-162 |
|  |  | FCA026-R | TGTACACGCACCAAAAACAA |  |  |
|  | FCA096 | FCA096-F | CACGCCAAACTCTATGCTGA | FAM | 96-235 |
|  |  | FCA096-R | CAATGTGCCGTCCAAGAAC |  |  |

| **Multiplex** | **Locus_ID** | **Primer** |  | **Label** | **Allele size** |
| --- | --- | --- | --- | --- | --- |
| M3 | FCA126 | FCA126-F | GCCCCTGATACCCTGAATG | VIC | 103-157 |
|  |  | FCA126-R | CTATCCTTGCTGGCTGAAGG |  |  |
|  | FCA043 | FCA043-F | GAGCCACCCTAGCACATATACC | NED | 106-146 |
|  |  | FCA043-R | AGACGGGATTGCATGAAAAG |  |  |
|  | FCA132 | FCA132-F | ATCAAGGCCAACTGTCCG | FAM | 127-161 |
|  |  | FCA132-R | GATGCCTCATTAGAAAAATGGC |  |  |
| M4 | FCA005 | FCA005-F | CCTAAGGAAACAGTAATCCTGGC | NED | 124-152 |
|  |  | FCA005-R | TGGCAGGCATACCAGGAT |  |  |
|  | FCA090 | FCA090-F | ATCAAAAGTCTTGAAGAGCATGG TGTTAGCTCATGTTCATGTGTCC | FAM | 82-118 |
|  |  | FCA090-R | TCCTGATGTGGCAGTTAAACCGCATGCCTTGAACCTTTCAT |  |  |
|  | FCA262 | FCA262-F | ATCTCTTCCATGGTGTGTGATG | VIC | 163-195 |
|  |  | FCA262-R | TACAGAATACTCCCCCCGC |  |  |
| M5 | FCA075 | FCA075-F | ATGCTAATCAGTGGCATTTGG | NED | 104-142 |
|  |  | FCA075-R | GAACAAAAATTCCAGACGTGC |  |  |
|  | FCA220 | FCA220-F | CGATGGAAATTGTATCCATGG | FAM | 202-224 |
|  |  | FCA220-R | GAATGAAGGCAGTCACAAACTG |  |  |
|  | FCA229 | FCA229-F | CAAACTGACAAGCTTAGAGGGC | VIC | 142-176 |
|  |  | FCA229-R | GCAGAAGTCCAATCTCAAAGTC |  |  |
|  | FCA441 | FCA441-F | ATCGGTAGGTAGGTAGATATAG | FAM | 127-173 |
|  |  | FCA441-R | GCTTGCTTCAAAATTTTCAC |  |  |

| **Multiplex** | **Locus_ID** | **Primer** |  | **Label** | **Allele size** |
| --- | --- | --- | --- | --- | --- |
| M6 | FCA094 | FAM094-F | TCAAGCCCCATTTTACCTTC | NED | 207-249 |
|  |  | FAM094-R | CACGCCAAACTCTATGCTGA |  |  |
|  | FCA105 | FAM105-F | TTGACCCTCATACCTTCTTTGG | FAM | 169-209 |
|  |  | FAM105-R | TGGGAGAATAAATTTGCAAAGC |  |  |
|  | FCA310 | FAM310-F | TTAATTGTATCCCAAGTGGTCA | VIC | 106-140 |
|  |  | FAM310-R | TAATGCTGCAATGTAGGGCA |  |  |

**Supplementary Figure**

**a2**

**a1**

**
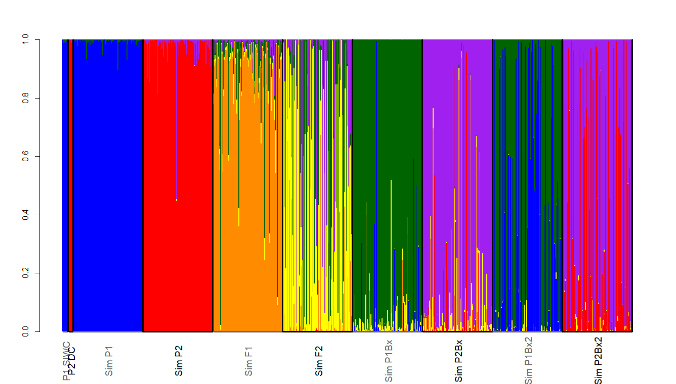
**
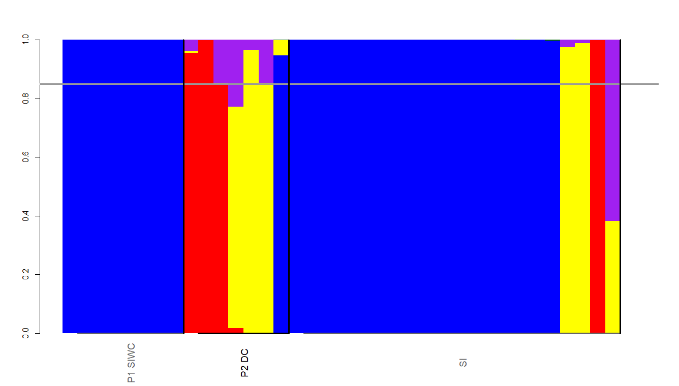


**b1**

**b2**


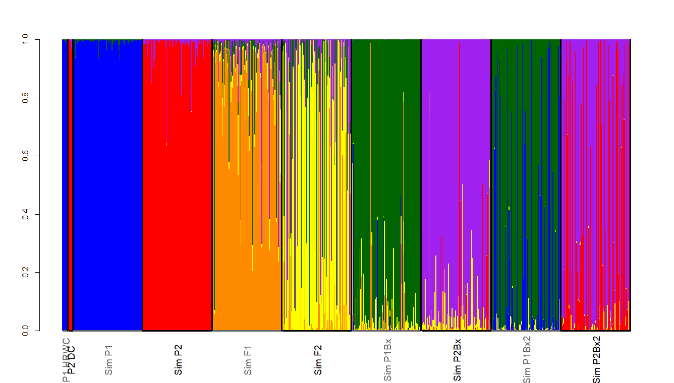

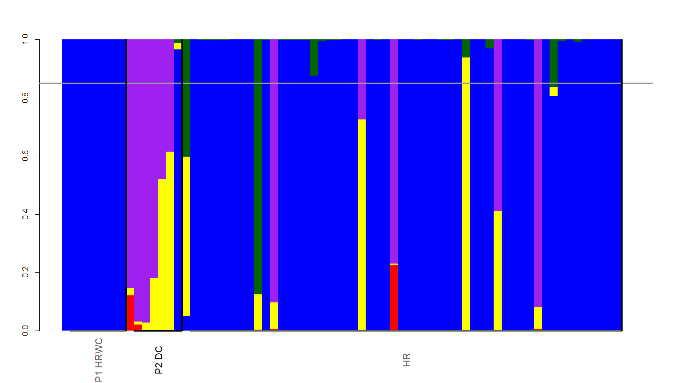


**c1**

**c2**


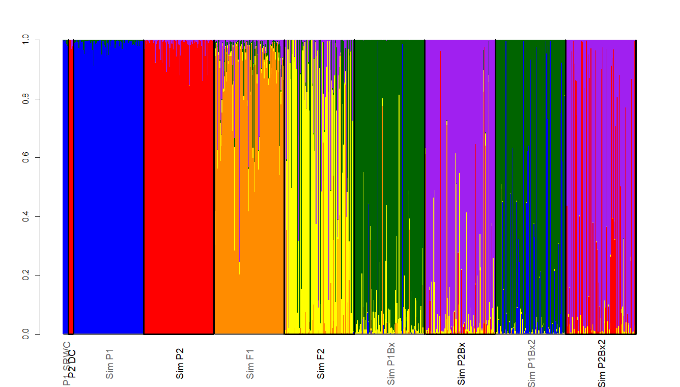

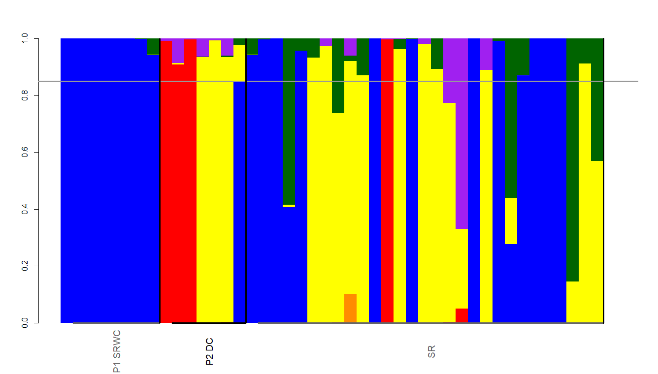


**Supplementary Figure 1. Detection of simulated hybrids with NewHybrids.** NewHybrids analysis of a Simulated genotypes with control samples from wildcats (P1) and domestic cats (P2) (8 genotypes each) and simulated F1, F2, F1 backcrosses to P1 (P1Bx), F1 backcrosses to P2 (P2Bx), P1Bx backcrosses to P1 (P1Bx2), and P2Bx backcrosses to P2 (P2Bx2) (100 simulated genotypes each) and Actual genotypes with control samples of P1 and P2. Each section represents figure of simulated genotype and below figure of actual genotype (a - Slovenia, b - Croatia; c- Serbia, due to small samples size Macedonian population was not included in analysis). A 0.95 cut-off was applied to all analyses as indicated by the solid grey line. Colours/shading reflect clustering based on NewHybrid settings, see Methods for details.


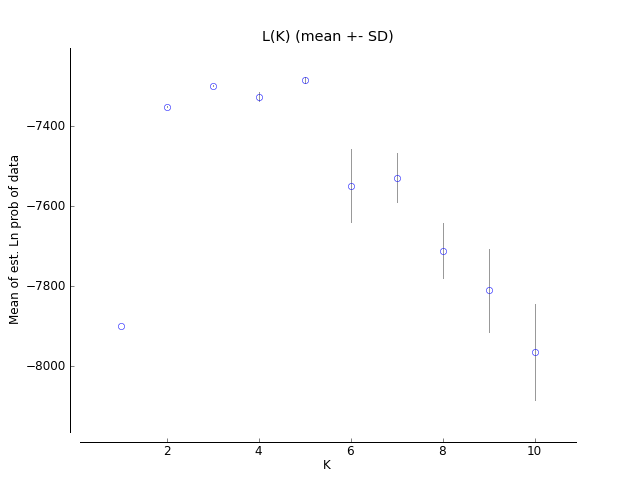

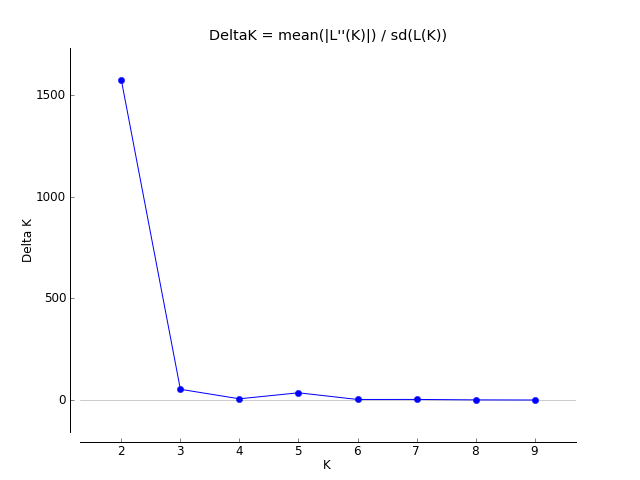


**b**

**a**

**Supplementary Figure 2.** **Graphic representation of the estimated probability of data for each K value.** Analysis parameters: Parameter Set - admixture model, allele frequencies correlated, POPFLAG=1, with ten replicates for each K value. a) the median and variance of the estimated probability value for each K value. It is possible to observe a plateau after K = 3; b) delta K calculated by the Evanno et al. (2005) method. The maximum value is observed at K = 2.

**b**

**a**

**
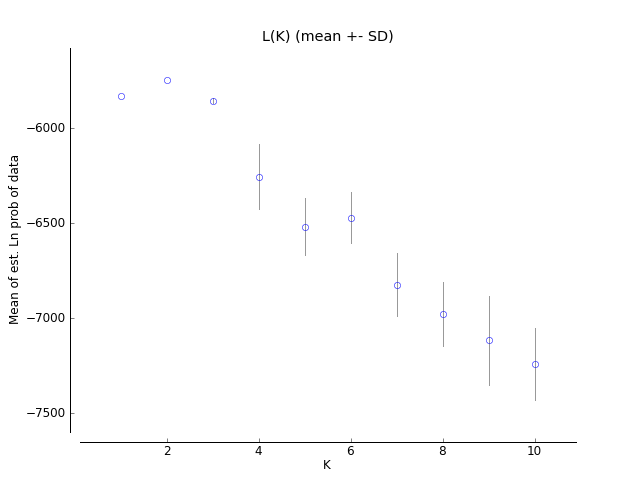

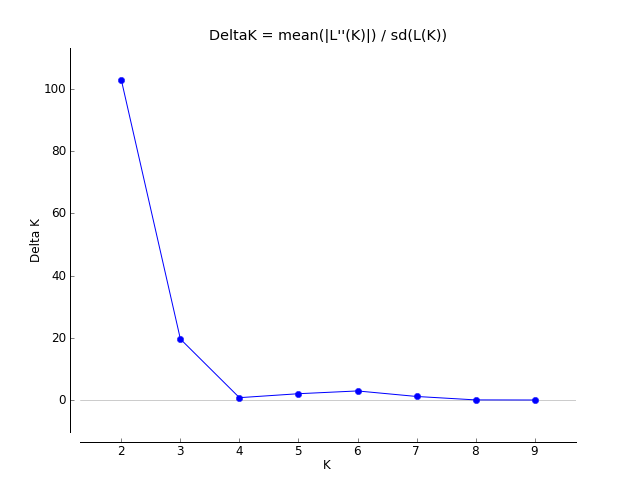
**

**Supplementary Figure 3.** **Graphic representation of the estimated probability of data for each K value.** Analysis parameters: Parameter Set - admixture model, allele frequencies correlated, POPFLAG=1, with ten replicates for each K value. a) the median and variance of the estimated probability value for each K value. It is possible to observe a plateau after K = 2. b) delta K calculated by the Evanno et al. (2005) method. The maximum value is observed at K = 2.

**
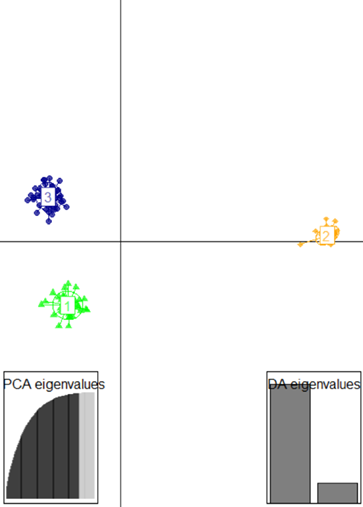
**

**Supplementary Figure 4.** **Discriminant analysis of principal components (DAPC)**. DAPC plots of genetic clusters of the wild and domestic cats (cluster 1 include SI and HR, cluster 2 include HRDc, cluster 3 include SR and MK populations). The axes represent the first two Linear Discriminants (LD). Each circle represents a cluster and each dot represents an individual. Numbers represent the different clusters identified by DAPC analysis.

**
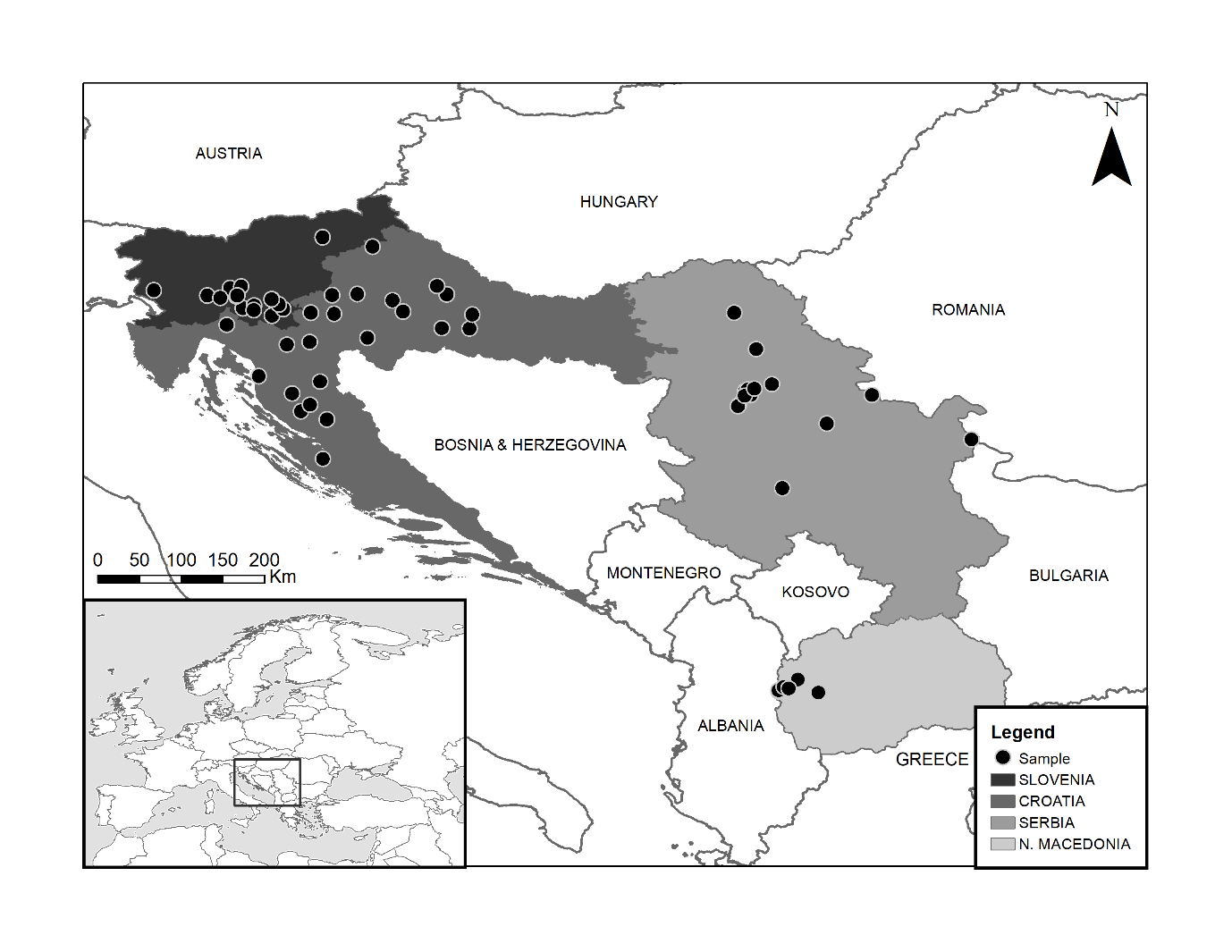
**

**Supplementary Figure 5.** **Sampling sites of European wildcats, included in the study.** Different shades indicate each country, (Slovenia (SI), Croatia (HR), Serbia (SR) and North Macedonia (MK) (see Table 1)). Black dots indicate the sample location. Insert shows the location of the area under study in Europe.
